# Levosimendan attenuates multiple organ injury and improves survival in peritonitis-induced septic shock: studies in a rat model

**DOI:** 10.1186/s13054-014-0652-4

**Published:** 2014-11-29

**Authors:** Cheng-Ming Tsao, Kai-Yi Li, Shiu-Jen Chen, Shuk-Man Ka, Wen-Jinn Liaw, Hsieh-Chou Huang, Chin-Chen Wu

**Affiliations:** Department of Anesthesiology, Taipei Veterans General Hospital and National Yang-Ming University, Taipei, Taiwan; Department of Anesthesiology, Tri-Service General Hospital, National Defence Medical Centre, Taipei, Taiwan; Department of Pharmacology, National Defence Medical Centre, Neihu PO Box 90048-504, Taipei, 114 Taiwan; Department of Nursing, Kang-Ning Junior College of Medical Care and Management, Taipei, Taiwan; Department of Physiology, National Defence Medical Centre, Taipei, Taiwan; Graduate Institute of Aerospace and Undersea Medicine, National Defence Medical Centre, Taipei, Taiwan; Department of Anesthesiology, Tungs’ Taichung MetroHarbor Hospital, Taichung, Taiwan; Department of Anesthesiology, Cheng-Hsin General Hospital, Taipei, Taiwan; Department of Pharmacology, Taipei Medical University, Taipei, Taiwan; Department of Anesthesiology and Pain Clinics, Cheng-Hsin Rehabilitation Medical Centre, 45, Cheng-Hsin St, Taipei, 112 Taiwan

## Abstract

**Introduction:**

The aim of this study was to investigate the effects of levosimendan on rodent septic shock induced by cecal ligation and puncture (CLP).

**Methods:**

Three hours after peritonitis-induced sepsis, male Wistar rats were randomly assigned to receive an intravenous infusion of levosimendan (1.2 μg/kg/min for 10 min and then 0.3 μg/kg/min for 6 h) or an equivalent volume of saline and vehicle (5% dextrose) solution.

**Results:**

The levosimendan-treated CLP animals had significantly higher arterial pressure and lower biochemical indices of liver and kidney dysfunction compared to the CLP animals (*P* < 0.05). Plasma interleukin-1β, nitric oxide and organ superoxide levels in the levosimendan-treated CLP group were less than those in CLP rats treated with vehicle (*P* < 0.05). In addition, the inducible nitric oxide synthase (iNOS) in lung and caspase-3 expressions in spleen were significantly lower in the levosimendan-treated CLP group (*P* < 0.05). The administration of CLP rats with levosimendan was associated with significantly higher survival (61.9% vs. 40% at 18 h after CLP, *P* < 0.05). At postmortem examination, the histological changes and neutrophil filtration index in liver and lung were significantly attenuated in the levosimendan-treated CLP group (vs. CLP group, *P* < 0.05).

**Conclusions:**

In this clinically relevant model of septic shock induced by fecal peritonitis, the administration of levosimendan had beneficial effects on haemodynamic variables, liver and kidney dysfunction, and metabolic acidosis. (1) Lower levels of interleukin-1β, nitric oxide and superoxide, (2) attenuation of iNOS and caspase-3 expressions, and (3) decreases of neutrophil infiltration by levosimendan in peritonitis-induced sepsis animals suggest that anti-inflammation and anti-apoptosis effects of levosimendan contribute to prolonged survival.

## Introduction

Despite better understanding of sepsis pathophysiology and improved advanced care in the past decade, the incidence and mortality of sepsis has substantially increased. In the presence of septic shock and associated multiple organ failure, mortality may approach 30 to 40% [1-3]. Considering that severe sepsis may potentially reduce regional tissue perfusion, the use of vasodilators to open up the microcirculation and improve tissue oxygenation in sepsis is reasonable [4,5]. Our previous study has also demonstrated that terbutaline, a β_2_-adrenoceptor agonist inducing peripheral vasodilation via the cyclic adenosine monophosphate pathway, reduces organ dysfunction and mortality in rats with severe sepsis [6].

The calcium sensitizer, Levosimendan (LS) enhances cardiac contractility independent from the adrenergic system by means of binding to the troponin C within cardiomyocytes [7]. In addition, LS causes venous, arterial and coronary vasodilation, probably by opening ATP-sensitive potassium channels (K_ATP_) in smooth muscle [8]. Experimental data show that LS improves cardiac performance and organ blood flow during experimental and human septic shock [9-12]. More importantly, both clinical and experimental studies manifest that LS has anti-inflammatory and anti-apoptotic properties in addition to its cardiovascular effects [13-15]. In a rat model of severe sepsis induced by cecal ligation and incision, inhaled LS has been found to reduce the releases of plasma IL-1β and IL-6 and expression of splenic caspase-3 [16].

Therefore, we examined the hypothesis that short-term infusion of LS administered after the establishment of peritonitis-induced sepsis would attenuate succeeding organ injury and increase survival in a more clinically relevant model of septic shock. In addition, we investigated the effect of LS on haemodynamics, pro-inflammatory cytokines, free radicals and caspase-3 expression, which may be associated with the organ dysfunction induced by sepsis.

## Materials and methods

All work in this study was approved by the Committee on the Ethics of Animal Experiments of National Defence Medical Centre (permit number IACUC-10-151), and the care and handling of the animals were in adherence to the National Institutes of Health Guidelines for ethical animal treatment. Male adult Wistar rats (280 to 350 g body weight), purchased from the National Laboratory Animal Centre of Taiwan, were kept on a 12-h light/dark cycle at a controlled temperature (21 ± 2°C) with free access to food and tap water.

### Surgical procedures

Rats were anaesthetised using intraperitoneal pentobarbital (40 to 50 mg/kg) and inhalation of isoflurane (0.5 to 1.0%). Unconscious rats were tested for sufficient depth of anaesthesia by pinching their toes. Polyurethane catheters for blood pressure measurement and drug administration were inserted into left carotid artery and right jugular vein, respectively. Subsequently, the catheters were positioned posteriorly, fixed to the back of the neck. The cannulated animals were allowed to recover to their normal condition overnight with standardised pellet food and tap water *ad libitum*.

After measurement of baseline haemodynamic analysis and collection of an arterial blood sample, the intraperitoneal sepsis was then induced by cecal ligation and puncture (CLP) using methods described previously [17]. Briefly, a 2-cm-long midline laparotomy was performed under sufficient anaesthesia. The exposed cecum was ligated with a 3-0 silk ligature just distal to the ileocecal valve, punctured twice with an 18-gauge needle. In the sham of control (SOP) group, cecal exposure was performed without any other manipulation. The cecum was then replaced into the abdomen and the abdominal incision was closed. All animals immediately received 0.9% NaCl solution (10 mL/kg subcutaneously) for intraoperative fluid loss.

All rats used in the study were kept in the small in-house animal facility of our institute to enable optimal monitoring: the overall health status was checked every 4 to 6 h for signs of distress. A subset of rats did not survive 18 h after induction of sepsis, and specifically, rats were euthanised only at the end of each experiment (at 18 h after CLP or sham surgery) or upon signs of imminent death (that is, unresponsive to external stimuli, inability to maintain an upright position, tremor and prolonged/deep hypothermia and/or agonal breathing) by using an overdose of pentobarbital (100 mg/Kg, given intravenously (i.v.)). Then, some tissue specimens of liver and lung were immediately exercised to analyze superoxide levels, western blotting and histological changes. In addition, the survival rate at 18 h in each group was analysed.

### Experimental protocol

Animals were divided into sham and CLP groups, and then i.v. infused with LS (1.2 μg/kg/minute for 10 minutes followed by 0.3 μg/kg/minute for 6 h), or the same volume of 9% saline and 5% dextrose solution in each group at 3 h after the sham and the CLP operation. LS (Orion Corporation, Espoo, Finland) was dissolved in 5% dextrose solution and its concentration was 0.01 mg/mL. Each arterial blood sample (0.8 mL) was collected at baseline (that is, time 0) and at specified times (that is, at 3, 9, and 18 h after CLP or sham surgery). An equal volume of sterile saline was used to immediately replace each volume of withdrawn blood.

### Measurement of haemodynamic parameters

The arterial catheter was connected to a pressure transducer (P23ID, Statham, Oxnard, CA, USA) for the measurement of phasic blood pressure and heart rate, which were displayed on a polygraph recorder (MacLab/4e, AD Instruments Pty Ltd., Castle Hill, Australia). The changes in haemodynamics were recorded at 0, 3, 9 and 18 h after CLP or sham surgery. After recording haemodynamic parameters at each time point, animals were intravenously given a norepinephrine (NE) bolus (1 μg/kg) to examine their vasopressor responses [18-20]. In order to normalise all results of vasopressor responses to their baseline values in all groups, we calculated the values at time 0 of each group as 100%.

### Quantification of organ function and injury

Some arterial blood (180 μL) was used to analyse the levels of pH, carbon dioxide tension (PaCO_2_), bicarbonate (HCO_3_^-^), base excess and potassium concentration by an arterial blood gas analyzer (AVL OPTI Critical Care Analyzer, AVL Scientific Corp., Roswell, GA, USA). Blood glucose was analysed by a One-Touch II blood glucose monitoring system (Lifescan Inc., Milpitas, CA, USA) with 10 μL of whole blood. The remaining blood was then immediately centrifuged at 7,500 *g* for 2 minutes to obtain the plasma. Plasma (80 μL) was used to analyse the biochemical parameters of liver and kidney function. Liver function was assessed by measuring plasma levels of aspartate aminotransferase (AST) and alanine aminotransferase (ALT), and renal function was assessed by plasma levels of blood urea nitrogen (BUN) and creatinine. In addition, plasma lactate dehydrogenase (LDH) was measured to evaluate the extent of organ injury. All of these biochemical parameters were analysed by Fuji DRI-CHEM 3030 (Fuji Photo Film Co., Ltd., Tokyo, Japan).

### Measurement of plasma nitric oxide concentrations

The plasma samples (30 μL) were used to measure plasma nitric oxide (NO) concentration, which in the study is actually depicted as the total nitrite and nitrate concentration in plasma. The nitrite/nitrate concentrations in all samples were measured using chemiluminescence, as previously described [21,22]. The amounts of nitrate in the plasma were measured by adding a reducing gent (0.8% VCl_3_ in 1 N HCl) to the purge vessel to convert nitrate to NO · , which was stripped from the plasma by using helium purge gas. The NO · was then drawn into a nitric oxide analyzer (Sievers 280 NOA; Sievers Inc., Boulder, CO, USA). Nitrate concentrations were calculated by comparison with standard solutions of sodium nitrate (Sigma Chemical Co., St Louis, MO, USA).

### Measurement of plasma IL-1β concentrations

The plasma samples (150 μL) were used to measure the plasma IL-1β in duplicate with an enzyme-linked immunoadsorbent assay kit (R&D Systems, Inc., Minneapolis, MN, USA) according to the manufacturer’s instructions.

### Measurement of superoxide production

Superoxide production was measured by lucigenin-enhanced chemiluminescence as previously described [23,24]. At the end of study, the animals were exsanguinated and sacrificed with overdose pentobarbital and some tissue specimens were immediately isolated and removed for analysis. The thoracic aorta was carefully trimmed of extravascular tissues and then cut into rings of 5-mm width. The liver, pancreas and spleen tissues (5 × 5 mm) were cleared of blood and transferred to scintillation plates. These scintillation plates containing Krebs-Hepes buffer with 1.25 mM lucigenin (final volume of 250 μL) were placed into a microplate luminometer (Hidex Microplate Luminometer, Turku, Finland). Counts were obtained in duplicate for all tissues, which then were dried for 24 h. The results were expressed as counts per sec (in each mg of dry tissue).

### Western blot analysis

After euthanasia, the lung and spleen were obtained and frozen at -80°C before assay. Frozen samples were thawed and homogenised on ice for protein assay (Bio-Rad Laboratories, Hercules, CA, USA), as previously described [17]. For western blotting, supernatants of tissue homogenates (100 μg total protein) were separated on a 10% (for inducible nitric oxide synthase (iNOS)) and 15% (for caspase-3) polyacrylamide gel and transferred on to a nitrocellulose membrane (Hoeffer, CA, San Francisco, USA). After blocking for 1.5 h at room temperature (5% skimmed milk in Tris-buffered saline containing 0.1% Tween 20), the membrane was incubated overnight (4°C) with polyclonal anti-mouse iNOS antibody (BD Transduction Laboratories, Lexington, KY, USA) or polyclonal anti-rabbit cleaved caspase 3 antibody (Cell Signaling Technology, Danvers, CO, USA) at a 1:1,000 dilution in blocking buffer followed by a horseradish-peroxidase-coupled secondary antibody (BD Transduction Laboratory) for 1 h at room temperature at a 1:5,000 dilution for iNOS and 1:3,000 for caspase-3. The immunoreactive proteins were visualised with the enhanced peroxidase/luminol chemiluminescence reaction kit (Amersham Pharmacia Biotech, Little Chalfont, UK) followed by exposure to radiographic film. The blots were then stripped and incubated with anti-β-actin antibody (diluted 1:3,000; BD Transduction Laboratory) to ensure equal loading. The ratios of the bands are shown.

### Histological assessment

Specimens of lung, liver and spleen were harvested and immediately fixed in 10% formaldehyde for more than 24 h. The fixed tissues were dehydrated in graded ethanol and embedded in paraffin. Each paraffin block was processed into 4 μm-thick slices that were stained with hematoxylin and eosin. This histological alteration was quantitatively analysed as indexes of the severity of polymorphonuclear neutrophil (PMN) infiltration and apoptosis in three animals from each group. Apoptotic cells were identified by the characteristic morphology of nuclear fragmentation (karyorrhexis) and cell shrinkage with condensed nuclei (pyknosis) [25]. The indexes were scored 0 (minimal) to 4 (maximal), determined by counting the numbers of PMN and apoptotic cells in 10 randomly selected high-power fields evaluated by a pathologist in a blinded fashion.

### Statistical analysis

The data are presented as mean ± standard error of the mean (SEM) of n determinations, where n represents the number of animals studied. The significance of differences in the measured values between groups was analysed using one-way analysis of variance (ANOVA) or two-way (time and group) ANOVA for repeated measures, followed by Bonferroni correction as a post hoc test. The scores for neutrophil infiltration and apoptosis were compared by the Mann-Whitney *U*-test. The chi-square test was used to evaluate the effect of treatment on survival rates. A *P*-value <0.05 was considered to be statistically significant.

## Results

### Systemic haemodynamic parameters

The baseline values of the haemodynamic parameters were comparable in all groups as shown in Table 1. CLP led to a significantly substantial attenuation in systolic blood pressure, diastolic blood pressure and pulse pressure at 18 h (P <0.05, versus the SOP group), and there was no significant difference between CLP and CLP + vehicle (Veh) groups. The treatment of CLP rats with LS significantly prevented severe hypotension and increased pulse pressure at 18 h (P <0.05, versus CLP + Veh). In addition, the SOP rats treated with LS had significantly increased pulse pressure at 18 h compared to the SOP rats treated with saline.

**Table 1:** **Changes in haemodynamic parameters**

**Variables**	**Time point**	**SOP (n = 10)**	**SOP + LS (n = 6)**	**CLP (n = 10)**	**CLP + Veh (n = 6)**	**CLP + LS (n = 10)**
Heart rate, beats/minute	0 h	388 ± 12	367 ± 13	334 ± 7	350 ± 15	369 ± 8
	3 h	413 ± 12	392 ± 16	444 ± 11*	461 ± 12*	474 ± 12*
	9 h	395 ± 10	382 ± 16	472 ± 11*	488 ± 20*	518 ± 13*
	18 h	388 ± 11	404 ± 20	492 ± 17*	499 ± 14*	478 ± 8*†
Systolic pressure, mmHg	0 h	140.3 ± 2.1	141.4 ± 3.3	133.7 ± 2.6	147 ± 4.9	139 ± 2.9
	3 h	146.6 ± 2.6	144.8 ± 1	139.8 ± 2.7	146.8 ± 6.1	141.8 ± 2.6
	9 h	148.1 ± 2.9	142.7 ± 1.9	124.7 ± 2.6*	130 ± 6.8	133.8 ± 3.4
	18 h	140.4 ± 3.2	146 ± 2.3	87.4 ± 5.1*	88 ± 5.1*	126.7 ± 4.1†
Diastolic pressure, mmHg	0 h	106.5 ± 2.6	103.9 ± 3.3	99.8 ± 2.4	111.9 ± 3.9	104.2 ± 2.5
	3 h	112.5 ± 2.3	108.2 ± 2.2	109.9 ± 2.5	118.4 ± 6.6	112.3 ± 2.7
	9 h	112.6 ± 3.5	104.6 ± 3	102.7 ± 2.6*	108.3 ± 5.8	96 ± 3.5
	18 h	105.8 ± 3.6	100.3 ± 4.4	66.7 ± 4*	69.5 ± 3.8*	83.8 ± 4.3*†
Pulse pressure, mmHg	0 h	33.8 ± 0.9	37.5 ± 1.5	33.9 ± 1.1	35.1 ± 2.4	35.3 ± 0.9
	3 h	34.1 ± 0.9	36.7 ± 1.6	30 ± 1.3	28.2 ± 2.3	29.5 ± 0.8
	9 h	35.5 ± 0.7	38.1 ± 2.2	22 ± 1*	21.6 ± 2*	37.9 ± 2.7†
	18 h	34.6 ± 0.9	45.7 ± 2.9*	20.7 ± 1.8*	18.6 ± 2.6*	42.9 ± 1.9*†
Pressor response to norepinephrine, %	0 h	100 ± 0	100 ± 0	100 ± 0	100 ± 0	100 ± 0
	3 h	90.8 ± 4.1	94.5 ± 4	40.8 ± 5.2*	31.1 ± 3.1*	33.4 ± 3.3*
	9 h	105 ± 3	96.4 ± 5.4	33.7 ± 3.1*	29.1 ± 2.5*	33.9 ± 2.8*
	18 h	103 ± 3.2	94.7 ± 2.6	21.9 ± 1.8*	19.5 ± 1.2*	31.1 ± 1.5*†

Depicted are changes in different groups of SOP (n = 10), SOP + LS (n = 6), CLP (n = 10), CLP + Veh (n = 6) and CLP + LS (n = 12). Data are expressed as mean ± standard error of the mean. *P <0.05, all groups versus SOP; ^†^ *P* <0.05, CLP + LS versus CLP + Veh. CLP, cecal ligation and puncture; LS, Levosimendan; SOP, sham of operation; Veh: vehicle (5% dextrose solution).

CLP led to a significant and sustained increase in heart rate at 3 to 18 h after CLP (*P* <0.05, versus SOP group; Table 1), and there was no difference between CLP and CLP + Veh groups. The treatment of CLP rats with LS significantly prevented the CLP-induced tachycardia at 18 h (P <0.05, versus CLP + Veh). The SOP rats treated with saline or LS exhibited stable haemodynamic conditions during the experimental period and there was no significant difference among groups.

CLP led to a significantly substantial attenuation in pressor responses to norepinephrine at 18 h (*P* <0.05, versus SOP group; Table 1), and there was no significant difference between the CLP and the CLP + Veh groups. However, LS administration significantly prevented the decrease of pressor responses to norepinephrine in the CLP group at 18 h. The SOP group treated with saline or LS exhibited stable haemodynamic conditions during the experimental period and there was no significant difference among groups.

### Organ injury/dysfunction

**Figure 1:**
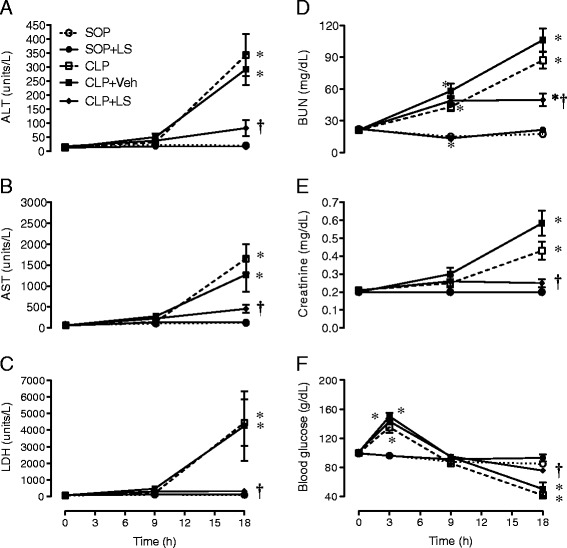
**Progress of plasma (A) alanine aminotransferase (ALT), (B) aspartate aminotransferase (AST), (C) lactate dehydrogenase (LDH), (D) blood urea nitrogen (BUN), (E) creatinine, and (F) blood glucose during the experimental period.** Depicted are the changes in plasma biochemical parameters in different groups of sham operation (SOP, n = 10), SOP plus levosimendan administration (SOP + LS, n = 6), cecal ligation and puncture (CLP, n = 10), CLP plus vehicle treatment (CLP + Veh, n = 6) and CLP plus levosimendan administration (CLP + LS, n = 12). Data are expressed as mean ± standard error of the mean. *P <0.05, all groups versus SOP. ^†^ *P* <0.05, CLP + LS versus CLP + Veh.

Baseline values of plasma markers of biochemical parameters were not significantly different among groups (Figure 1). No significant changes in these parameters were observed during the experimental period in the SOP group treated with saline or LS, and there was no significant difference between the CLP and the CLP + Veh groups.

Significant increases in plasma levels of ALT, AST, LDH and creatinine at 18 h, and BUN at 9 and 18 h were found after CLP compared to the SOP group (*P* <0.05, Figure 1A-C and E). The rises in plasma AST, ALT, LDH, BUN and creatinine levels at 18 h after CLP were attenuated by LS (*P* <0.05 versus CLP + Veh).

The CLP surgery induced a biphasic change in blood glucose, that is, hyperglycemia at the early stage (3 h) and hypoglycemia at the late stage (18 h) (*P* <0.05 versus SOP group; Figure 1F). However, the treatment of CLP rats with LS significantly ameliorated the hypoglycemia at 18 h (*P* <0.05, versus CLP + Veh), yet the level was below the baseline and the SOP values.

### Blood gas parameters and blood potassium concentration

There was no statistical difference in blood gas parameters in SOP rats, and there was no statistical difference in blood pH level among groups during the experimental period (Table 2). However, the rats treated with CLP for 18 h had significant decreases in PaCO_2_, HCO_3_^-^ and base excess at 9 and 18 h (*P* <0.05, versus SOP group; Table 2), indicating respiratory compensation for metabolic acidosis. The compensated metabolic acidosis induced by CLP at 18 h was ameliorated by the treatment with LS (*P* <0.05, versus CLP + Veh; Table 2). However, there was no significant difference between CLP and CLP + Veh groups.

**Table 2:** **Changes in blood gas parameters and potassium concentration**

**Variables**	**Group**	**SOP**	**SOP + LS**	**CLP**	**CLP + Veh**	**CLP + LS**
pH	0 h	7.6 ± 0.01	7.5 ± 0.01	7.6 ± 0.02	7.6 ± 0.01	7.6 ± 0.01
	9 h	7.5 ± 0.01	7.5 ± 0.01	7.6 ± 0.01	7.6 ± 0.02	7.6 ± 0.02
	18 h	7.6 ± 0.01	7.6 ± 0.01	7.5 ± 0.01	7.5 ± 0.03	7.6 ± 0.01
PaCO_2_, mmHg	0 h	34 ± 1.7	37 ± 2.3	32 ± 1.7	34 ± 0.4	35 ± 0.7
	9 h	36 ± 1	37 ± 1.8	22 ± 0.9*	24 ± 1.4*	24 ± 1.7*
	18 h	31 ± 1.2	32 ± 1.4	20 ± 1.3*	21 ± 1.9*	28 ± 1.4†
HCO_3_^-^, mM	0 h	30 ± 0.7	31.1 ± 1.1	27.9 ± 0.9	30 ± 0.7	29.9 ± 0.6
	9 h	29.4 ± 0.6	29.4 ± 1.1	20.8 ± 0.8*	22.1 ± 1.1*	23.2 ± 0.8*
	18 h	27.6 ± 0.7	28.5 ± 1.4	16.7 ± 1*	15.4 ± 1.2*	24.5 ± 1†
Base excess, mM	0 h	7.8 ± 0.5	8.4 ± 0.7	7.3 ± 0.2	7.9 ± 0.8	7.5 ± 0.5
	9 h	6.7 ± 0.5	6.7 ± 0.7	1.2 ± 0.7*	2.2 ± 1.1*	3.6 ± 0.5*
	18 h	5.4 ± 0.5	6.7 ± 1.1	-3.2 ± 0.8*	-5.5 ± 1.3*	3.2 ± 0.9*†
Potassium, mM	0 h	3.7 ± 0.1	3.9 ± 0.1	3.8 ± 0.1	3.8 ± 0.1	3.9 ± 0.1
	9 h	3.6 ± 0.1	4 ± 0.1	3.9 ± 0.1	3.9 ± 0.2	3.9 ± 0.1
	18 h	3.6 ± 0.1	3.7 ± 0.2	4.9 ± 0.2*	5.2 ± 0.4*	3.8 ± 0.1†

Depicted are the changes in different groups of SOP (n = 10), SOP + LS (n = 6), CLP (n = 10), CLP + Veh (n = 6) and CLP + LS (n = 12). Data are expressed as mean ± standard error of the mean. *P <0.05 all groups versus SOP; ^†^ *P* <0.05, CLP + LS versus CLP + Veh. CLP: cecal ligation and puncture; LS: Levosimendan; SOP: sham of operation; Veh: vehicle (5% dextrose solution).

There was no significant difference in blood potassium concentration in the SOP rats (Table 2). However, the rats treated with CLP for 18 h had significant increases in potassium concentration at 18 h (*P* <0.05, versus the SOP group; Table 2), and there was no significant difference between the CLP and the CLP + Veh groups. The hyperkalemia induced by CLP at 18 h was ameliorated by the treatment with LS (*P* <0.05 versus CLP + Veh; Table 2).

### Plasma nitrite/nitrate levels

**Figure 2:**
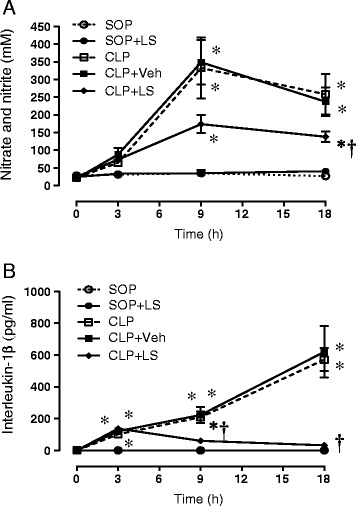
**Progress of plasma (A) nitric oxide (NO) and (B) interleukin-1β (IL-1β) levels during the experimental period.** Depicted are the changes of plasma NO and IL-1β levels in different groups of sham operation (SOP, n = 10), SOP plus Levosimendan administration (SOP + LS, n = 6), cecal ligation and puncture (CLP, n = 10), CLP plus vehicle treatment (CLP + Veh, n = 6) and CLP plus Levosimendan administration (CLP + LS, n = 12). Data are expressed as mean ± standard error of the mean. *P <0.05, all groups versus SOP; ^†^ *P* <0.05, CLP + LS versus CLP + Veh.

The basal plasma level of nitrite/nitrate was not significantly different among groups (Figure 2A). The CLP surgery led to a significant increase in plasma nitrite/nitrate level (*P* <0.05 versus the SOP group), which reached a plateau at 9 h, and there was no difference between the CLP and the CLP + Veh groups. The treatment of CLP rats with LS significantly inhibited the CLP-induced increase in plasma nitrite/nitrate level at 9 and 18 h (*P* <0.05 versus CLP + Veh). In the SOP group, no significant increase in plasma nitrite/nitrate levels was detectable during the experimental period. The treatment of SOP rats with LS alone had no significant effect on plasma level of nitrite/nitrate.

### Plasma IL-1β Levels

The basal plasma level of IL-1β was not significantly different between any of the experimental groups studied (Figure 2B). The CLP surgery led to significant increases in plasma IL-1β levels, and reached a peak at 18 h (*P* <0.05 versus the SOP group), and there was no significant difference between the CLP and the CLP + Veh groups. The treatment of CLP rats with LS significantly inhibited the CLP-induced increases in plasma IL-1β levels at 18 h (*P* <0.05 versus CLP + Veh). In the SOP group, no significant increases in plasma IL-1β levels were detectable during the experimental period. The treatment of SOP rats with LS alone, however, had no significant effect on plasma level of IL-1β.

### Organ superoxide levels

**Figure 3:**
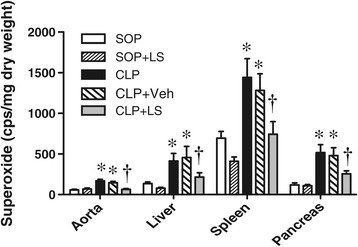
**Superoxide levels of aorta, liver, spleen and pancreas at 18 h after surgery.** Depicted are the changes in different groups of sham operation (SOP, n = 10), SOP plus Levosimendan administration (SOP + LS, n = 6), cecal ligation and puncture (CLP, n = 10), CLP plus vehicle treatment (CLP + Veh, n = 6) and CLP plus Levosimendan administration (CLP + LS, n = 12). Data are expressed as mean ± standard error of the mean. *P <0.05, all groups versus SOP, ^†^ *P* <0.05, CLP + LS versus CLP + Veh.

The basal production of superoxide was detectable in aorta, liver, spleen and pancreas in SOP rats at the end of the experiment (Figure 3). A significant increase in the superoxide levels of these organs was observed in CLP rats (*P* <0.05 versus the SOP group), and there was no significant difference between the CLP and the CLP + Veh groups. However, the treatment of CLP rats with LS significantly inhibited the production of superoxide (*P* <0.05 versus CLP + Veh), whereas LS itself had no significant effect on the change of superoxide level in SOP rats.

### iNOS protein and caspase-3 expression

**Figure 4:**
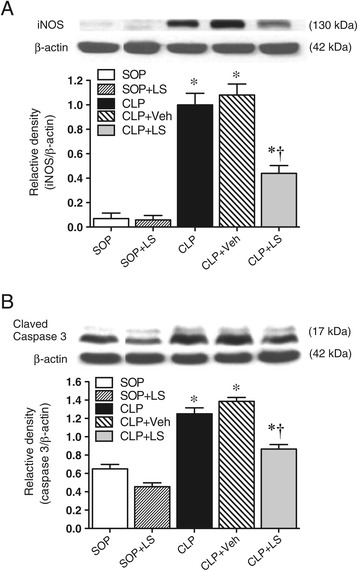
**Expressions of (A) lung inducible nitric oxide (NO) synthase (iNOS) and (B) spleen-cleaved caspase 3.** Depicted are the expressions of lung iNOS and spleen-cleaved caspase 3 at the end of the study in different groups of sham operation (SOP, n = 3), SOP plus Levosimendan administration (SOP + LS, n = 3), cecal ligation and puncture (CLP, n = 5), CLP plus vehicle treatment (CLP + Veh, n = 5) and CLP plus Levosimendan administration (CLP + LS, n = 5). Data are expressed as mean ± standard error of the mean. *P <0.05, all groups versus SOP, ^†^ *P* <0.05, CLP + LS versus CLP + Veh.

The protein expression of iNOS protein was undetectable in lung homogenates obtained from both SOP rats (Figure 4A). A significant induction of iNOS protein was observed in lung homogenates from the CLP rats (*P* <0.05 versus the SOP group), and there was no significant difference between CLP and CLP + Veh groups. The treatment of CLP rats with LS significantly reduced the expression of iNOS protein in the lung (*P* <0.05 versus CLP + Veh).

The protein expression of cleaved caspase-3 protein was undetectable in spleen homogenates obtained from both SOP rats (Figure 4B). A significant induction of cleaved caspase-3 protein was observed in spleen homogenates from the CLP rats (*P* <0.01 versus the SOP group), and there was no significant difference between the CLP and the CLP + Veh groups. The treatment of CLP rats with LS significantly reduced the expression of cleaved caspase-3 protein in the spleen (*P* <0.05 versus CLP + Veh).

### Histological studies

**Figure 5:**
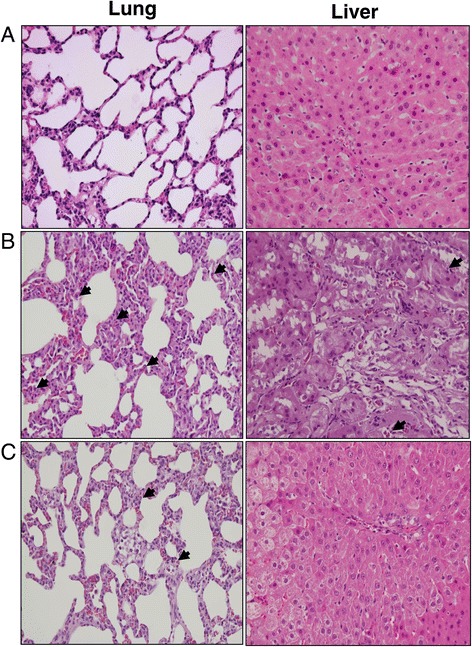
**Histopathological studies of lung and liver.** Light microscopy showed lung and liver sections of rats in groups of **(A)** sham operation, **(B)** cecal ligation and puncture (CLP), and **(C)** CLP plus Levosimendan administration. Sections were stained with haematoxylin and eosin. Arrows represent polymorphonuclear neutrophil infiltration in lung and liver. Each is shown at 400 × (original magnification).

**Figure 6:**
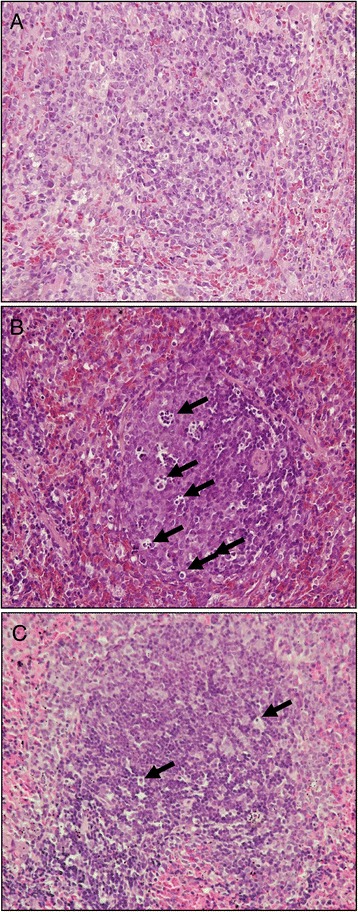
**Histopathological studies of spleen.** Light microscopy showed spleen sections of rats in groups of **(A)** sham operation, **(B)** cecal ligation and puncture, and **(C)** CLP plus Levosimendan administration. Sections were stained with haematoxylin and eosin. Arrows represent apoptosis in the spleen. Each is shown at 400 × (original magnification).

At the end of the study, stained specimens from CLP rats revealed 1) increased interstitial edema and decreased alveolar spaces in the lung, and 2) increased interstitial edema and marked necrosis in the liver compared to those from the SOP group (Figure 5). However, the histopathological changes in these organs were improved after LS treatment. Light microscopy only showed a little infiltration or sequestration of PMNs in lung and liver from the SOP group, whereas overt infiltration of PMNs in these tissues was observed in CLP rats (.0 ± 0.6 in lung and 2.0 ± 0.6 in liver; *P* <0.05). There was no significant difference between the CLP and the CLP + Veh groups. However, in CLP rats treated with LS, the PMN infiltrations were significantly reduced (1.7 ± 0.3 in lung, and 0.0 in liver; *P* <0.05 versus CLP). In addition, light microscopy showed no apoptosis in the spleen from SOP rats (Figure 6), whereas severe apoptosis were found in this organ from CLP rats (3.7 ± 0.3 in spleen; *P* <0.05). However, the apoptosis index was significantly reduced in CLP rats treated with LS (2 ± 0; *P* <0.05 versus CLP).

### Survival rate

No mortality was observed within 18 h in the SOP rats (survival rate = 100%). The 9-h and 18-h survival rates of CLP animals were 64% (that is, 16/25 animals) and 40% (that is, 10/25 animals), respectively (Table 3), while the 9-h and 18-h survival rates of CLP + Veh animals were 64.7 (that is, 11/17 animals) and 41.1% (that is, 7/17 animals), respectively. There was no significant difference in survival rate between CLP and CLP + Veh groups. In contrast, the rats treated with LS had higher survival rates of 71.4% (that is, 15/21 animals) at 9 h and 61.9% (that is, 13/21 animals) at 18 h after CLP. Thus, LS significantly increased the 18-h survival rate of CLP rats (*P* <0.05 versus CLP + Veh). Because of clots or kinking of the arterial catheter, blood and data could not be collected in some of rats in each group that survived the full 18 h of the experiment.

**Table 3:** **Efffects of Levosimendan (LS) on the survival rate in septic rats duing the pexperimental period**

**Groups**	**Survival rats (%)**	
	**9 h**	**18 h**
SOP	100	100
SOP + LS	100	100
CLP	64*	40*
CLP + Veh	64.7*	41*
CLP + LS	71.4*†	61.9*†

Rats underwent sham operation (SOP, n = 10), SOP and Levosimendan administration (SOP + LS, n = 6), cecal ligation and puncture (CLP, n = 25), CLP and vehicle treatment (CLP + Veh, n = 17) or CLP and Levosimendan administration (CLP + LS, n = 21). *P <0.05, CLP versus SOP; ^†^ *P* <0.05, CLP + LS versus CLP + Veh.

## Discussion

The major findings are that an intravenous infusion of LS: 1) increased arterial blood pressure and pressor response to the NE, 2) reduced plasma levels of biochemical parameters, 3) attenuated metabolic acidosis, and 4) prevented histopathological changes in CLP-treated rats. This study demonstrated that the LS application improved survival in sepsis as a consequence of reducing multiple organ injury. In addition, LS acted by inhibiting plasma IL-1β and NO production, attenuating superoxide formation, and suppressing iNOS and cleaved caspase-3 expression.

Clinically, LS is dissolved in a glucose 5% solution and administered preferably by continuous intravenous infusion. An interesting observation in the present study was the fact that 6-h infusion of LS administered 3 h later after CLP procedure had the capability of improving survival at the end of the 18-h experiment, while the infusion of vehicle (that is, 5% dextrose in this study) did not change the survival of CLP-treated rats. Furthermore, Scheiermann *et al*. found that a single dose of intravenous LS could prolong survival for more than 3 h after the onset of sepsis [16]. The elimination half-life of LS is approximately 1 h, however, the active LS metabolite OR-1896 has a longer half-life of 75 to 80 h [26]. Thus, OR-1896 may also potentially account for the protective effects of LS in our study.

The animal model of CLP developed hypotension and tachycardia as typically seen in human septic shock [27,28]. In the present study, the infusion of LS increased pulse pressure in sham- and CLP-treated rats, suggesting this is due to its positive inotropic effects. Vasodilation induced by activation of the K_ATP_ channel is another major property of LS. However, the LS dosage used in our study did significantly raise diastolic blood pressure but did not aggravate hypotension induced by CLP. Thus, this may be due to a positive inotropic effect outweighing the vasodilating component of LS. The administration of CLP rats with LS also attenuated sepsis-induced hypo-reactivity to NE possibly, due to its increasing effect on calcium sensitisation. It has been shown that sepsis-induced hypo-reactivity to norepinephrine is associated with NO-derived peroxynitrite [29-31]. Therefore, LS-decreased plasma NO levels in sepsis may also contribute to the improvement of vascular dysfunction.

There is increasing evidence that the apoptotic mode of cell death in critically ill patients plays a pivotal role in the pathogenesis of the sepsis syndrome [32,33]. Apoptotic cell death can be induced by caspase-3 through the extrinsic death-receptor and intrinsic mitochondria pathway, which can be activated by diverse stimuli, including pro-inflammatory cytokines, reactive oxygen species and NO [34,35]. Together with increased amounts of NO that are produced by the iNOS, superoxide forms the highly reactive peroxynitrite that causes irreversible damage to proteins, causing mitochondrial dysfunction and organ failure [32]. Our present study demonstrated that IL-1β, NO and superoxide production and iNOS expression were increased in CLP-induced septic rats, which were attenuated by LS administration. Furthermore, decreased apoptosis, as determined by the cleaved caspase-3 protein expression, was observed in the spleen of the CLP + LS group compared to the CLP + Veh group, indicating LS has an anti-apoptosis effect in the spleen [36].

Furthermore, LS administration decreased the inflammatory infiltration by neutrophils in vital organs such as liver and lung. Such neutrophil infiltration can lead to vascular dysfunction as well as parenchymal cell injury [37]. This indicates that LS could prevent organ injury in sepsis by its antioxidant and anti-inflammatory properties. Torraco *et al*. also reveal that LS protects mitochondria from the oxidative stress in patients suffering with septic shock [38]. These results suggest that beneficial effects of LS administration on biomarkers of oxidative stress, inflammation, tissue injury, and apoptosis were further strengthened by the favorable outcome in the study group, that is, LS-treated CLP animals had about 22% survival benefit over CLP controls.

On the other hand, splanchnic hypoperfusion and subsequent mucosal ischaemia result in increased inflammation, gut permeability and bacterial translocation, further exacerbating multiple organ dysfunction induced by sepsis [39]. In addition, hepatic and renal dysfunction may lead to the systemic release of inflammatory toxins, which further worsen tissue injury. Much importance has recently been attributed to tissue oxygenation in sepsis, and it has been suggested that vasodilators could be used therapeutically to increase the microcirculation and improve tissue oxygenation [40,41]. Earlier studies demonstrate that LS increases sublingual oxygenation and splanchnic perfusion during clinical septic shock [10,42]. In addition, LS increases portal blood flow, intestinal mucosal oxygenation and vascular reactivity in a porcine model of sepsis, whereas AST is not significantly attenuated [12]. However, biochemical parameters of liver and renal injury and metabolic acidosis were attenuated after LS treatment in our present study. Furthermore, light microscopy showed that the histopathological changes in these tissues were also improved. It is assumed to be associated with opening of the mitochondria K_ATP_ channel, increasing perfusion of peripheral tissues and decreasing organ injury [43,44].

The current study has some limitations that need to be addressed. First, it was conducted in previously healthy animals under highly controlled circumstances, in contrast to the clinical setting in which patients often have underlying illness and co-morbidities. Second, we did not use antibiotics in order to avoid any influence of LS on the organ function in this study. Moreover, only one single intravenous dose of continuous LS infusion was used, and consequently, we cannot exclude the possibility that a larger dose could yield better histological results. Finally, it has been shown that the administration of drugs just after endotoxin administration and CLP procedure does not represent medical practice. In the current study, LS was given at 3 h after CLP, when sepsis seems to be developing or to have developed. However, the use of LS for a longer period of time after CLP might have different results. Therefore, these results cannot be directly directed to clinical use unless similar interventions have been taken in this sepsis model.

## Conclusions

The present findings support the hypothesis that LS improved survival, minimized histological changes and prevented sepsis-induced multiple organ dysfunction by its anti-inflammatory and anti-apoptosis properties, which involve the inhibition of IL-1β and superoxide production as well as the reduction of iNOS and caspase-3 expression to the affected tissues. Thus, our present study suggests that a single early dose of LS could be a highly promising agent for protecting tissues from oxidative stress and preventing organ dysfunction due to sepsis, but this hypothesis needs to be proved in further clinical studies.

## Key messages

- Multiple organ dysfunction occurs in rats with peritonitis-induced sepsis
- Levosimendan is protective in the treatment of peritonitis-induced sepsis with respect to survival
- Levosimendan improves haemodynamic variables, reduces organ injury, and attenuates metabolic acidosis in septic rats
- Levosimendan may have anti-inflammatory and anti-apoptosis properties, which involve the inhibition of IL-1β and superoxide production as well as the reduction of iNOS and caspase-3 expression to the affected tissues

## Abbreviations

ALT: alanine aminotransferase
ANOVA: analysis of variance
AST: aspartate aminotransferase
BUN: blood urea nitrogen
CLP: cecal ligation and puncture
IL-1β: interleukin-1β
iNOS: inducible nitric oxide synthase
LDH: lactate dehydrogenase
LS: Levosimendan
NO: nitric oxide
PMN: polymorphonuclear neutrophil
SOP: sham of operation
Veh: vehicle
